# Digital Ischemic Loss in Systemic Sclerosis

**DOI:** 10.1155/2010/130717

**Published:** 2010-08-24

**Authors:** Umaima Marvi, Lorinda Chung

**Affiliations:** ^1^Division of Immunology and Rheumatology, Stanford University School of Medicine, Stanford, CA 94305, USA; ^2^Division of Rheumatology, Palo Alto Veterans Affairs Health Care System, Palo Alto, CA 94304, USA

## Abstract

Digital ischemic loss is a cause of significant morbidity in patients with systemic sclerosis (SSc). Microvascular disease with intimal proliferation and luminal narrowing of small digital arteries, as well as macrovascular disease with narrowing or occlusion of larger digital arteries, contribute to the perfusion defects involved in digital ischemic loss. Immediate clinical evaluation and treatment are mandatory at the onset of critical digital ischemia to prevent digital loss. Hospitalization for medical therapies including intravenous prostacyclin therapy should be considered for all SSc patients who present with critical digital ischemia. Surgical interventions are typically reserved for patients who fail medical therapies and for those with late stage, necrotic tissue. This paper summarizes the current knowledge regarding the risk factors, pathogenesis, evaluation, and treatment of digital ischemic loss in SSc.

## 1. Introduction

Systemic sclerosis (SSc) is a disease of unknown etiology characterized by immune activation, tissue fibrosis, and vasculopathy. Peripheral vascular involvement manifesting as Raynaud's phenomenon (RP) affects almost all patients. In a subset of SSc patients, episodes of progressive digital ischemia can result in a digital ulcer—a denuded area with a defined border with loss of epithelialization and loss of epidermis and dermis. Sustained reduction in digital perfusion with impaired tissue viability can lead to critical ischemia, in some cases resulting in gangrene necessitating amputation. On a microvascular scale, episodes of digital ischemia are thought to be due to neuroendothelial imbalance of vasoconstriction and vasodilatation, structural abnormalities of the vasculature, and intravascular factors such as platelet activation, procoagulants, and oxidative stress. Recent studies on macrovascular disease in SSc suggest that ischemic demarcation and loss of digits occurs secondary to narrowing or occlusion of larger digital arteries (vessels of the palmar arch, radial, or ulnar artery) or medium-sized and large arteries in the lower extremities. This paper will summarize the current knowledge regarding the risk factors, pathogenesis, evaluation, and treatment of digital ischemic loss in SSc.

## 2. Clinical Burden

SSc has a prevalence of 1–50 cases per 100,000 people worldwide [1]. A retrospective review of the clinical status of 98 patients with SSc, seen between 1985 and 1990, showed that amputation of 1 or more digits due to ischemia occurred in 20.4% of the patients while 9.2% had multiple digit loss [2]. A more recent review of a prospective cohort from 2001 found that 28 (16%) of 171 SSc patients attending a hospital in the UK had at least one digital amputation and 73 (43%) had experienced at least one episode of severe digital ischemia as defined by requirement for intravenous vasodilator therapy, surgical debridement, and/or amputation [3]. In another larger cohort of 1168 SSc patients in the UK followed over an 18 month period, 17.4% were found to have complications related to severe digital vasculopathy including digital ulcers, critical digital ischemia, gangrene, or the need for digital sympathectomy. One-third of these patients were on some immunosuppressive treatment, three-fourths were receiving at least one vasodilator, and one-fifth were on antiplatelet agents. Sixteen percent had at least one digital ulcer and 12% required at least one hospitalization for intravenous prostanoid therapy over the 18 month study period. Furthermore, 1.6% of the total cohort developed critical digital ischemia, 1.4% developed digital gangrene, 1.1% underwent a sympathectomy, and 0.9% had an amputation during the 18 month period [4].

## 3. Risk Factors

Predictors of ischemic digital loss in patients with SSc have been identified from data collected from observational studies (Table 1). Patients with long-standing limited cutaneous SSc (lcSSc) have traditionally been thought to have more prominent vascular manifestations than patients with diffuse cutaneous disease (dcSSc) [5]. However, Denton and colleagues noted that in their analyses of a prospective cohort of 1168 patients with SSc, severe digital vasculopathy occurred in 27.5% of the patients with dcSSc versus 13% of the patients with lcSSc (*P* < .0001). There was no correlation between disease duration and severity of digital vasculopathy [4].

An initial observation by Herrick et al. found weakly positive anti-cardiolipin (aCL) antibodies in four of eight patients with SSc who had severe digital ischemia requiring amputation [6]. However, a follow-up retrospective analysis found that there was no difference in aCL positivity in SSc patients with severe ischemia (11/31) versus those without (16/37), or between those who had amputation (5/13) and those who had not (22/55). Instead, this study found an association between the presence of the anti-centromere antibody and severe peripheral ischemia. Seventeen of the 31 patients (55%) with severe ischemia were anti-centromere antibody positive compared with nine of 37 (24%) without ischemia (*P* = .01). There was also a trend for an association with the presence of the anti-topoisomerase antibody, in that six patients with severe ischemia had anti-topoisomerase antibodies compared with two patients without ischemia (*P* = .08). The authors concluded that complications from severe digital vasculopathy are more likely in patients with scleroderma specific antibodies [7]. 

Another review of a prospective cohort from the UK also found that anti-centromere antibody positivity is a predictor for amputation. Of 171 patients, 75% of whom had lcSSc, 37% were anti-centromere antibody positive and 16.4% were identified as having amputations. Of patients who had undergone amputations, 60.7% were anti-centromere antibody positive (OR 3.12). Smoking was also found to be an independent risk factor for amputation (OR 6.28 per pack per day, 95% CI 1.95 − 20.19) [3].

Other studies have also investigated whether smoking is a risk factor for poorer outcomes related to digital ischemia. Previously, Wigley et al. concluded that smoking was not a risk factor in 98 patients in their SSc cohort at a US tertiary care center [8]. However, an analysis from a database from the UK found that in their cohort of 101 SSc patients, current smokers were 3-4 times more likely than never-smokers to incur digital vascular complications. When adjusting for age, sex, and disease duration, current smokers were significantly more likely than never-smokers to have required debridement (OR 4.5, 95% CI 1.1–18.3) or admission for intravenous vasodilators (OR 3.8, 95% CI 1.1–12.9) [9]. 

A recent study investigated whether anti-beta2-glycoprotein I (anti-beta2-glycoprotein I antibodies) and aCL antibodies are correlated with macrovascular disease, including digital loss in SSc patients. Seventy five SSc patients with a history of ischemic digital loss were matched to 75 SSc patients without a history of digital loss. Anti-beta2-glycoprotein I antibodies was significantly more frequent in SSc patients with digital loss than in patients without digital loss: 27/75 (36%) in the digital loss group had these antibodies compared with 14/75 (19%) in the group without digital loss (*P* = .017). The IgA subtype showed the strongest association (OR 4.0, CI 1.1–14.2). However, there was no difference in aCL antibody frequency between the two groups, as has been previously reported. In addition, after adjusting for demographics, disease type, smoking status, and anti-centromere antibodies, anti-beta2-glycoprotein I antibodies positivity was significantly associated with active digital ischemia (OR 9.4, CI 3.5–25.4), elevated estimated right ventricular systolic pressure (RVSP) (OR 4.8, CI 1.0–11.4), and increased mortality (OR 2.9, CI 1.1–7.1). They also noted that patients with a history of ischemic digital loss have more severe pulmonary vascular disease with overall a higher RVSP, worse lung severity scores, and lower diffusing capacity of carbon monoxide (DLco) which might be contributing to the higher mortality. All in all, anti-beta2-glycoprotein I antibodies is likely important in scleroderma vascular disease; however, further research is necessary to determine whether these antibodies are directly involved in the pathogenesis of disease or represent an epiphenomenon [10]. 

 In addition, antibodies against novel autoantigens expressed in the cytotoxic lymphocyte granule pathway have been associated with the clinical phenotype of ischemic digital loss in SSc. Specifically, autoantibodies to granzyme B, a serine protease found in the cytoplasmic granules of cytotoxic T cells and natural killer cells, with an important role in inducing apoptosis and clearance of intracellular pathogens, has been found to be highly associated with the phenotype of ischemic digital loss. Investigators at Johns Hopkins University found that the sera from 16/19 (84.2%) lcSSc patients with ischemic digital loss immunoblotted for autoantigens to granzyme B compared with 6/15 (40%) lcSSc patients without ischemic digital loss (OR 8.0, CI 1.6–40.0). The risk of anti-granzyme B antibodies persisted even when controlling for the presence of antiphospholipid antibodies [11]. This in vivo recognition of granzyme B-generated autoantigen fragments in lcSSc patients with ischemic digital loss identifies a distinct clinical subset and needs further investigation.

## 4. Pathogenesis

The pathogenesis of SSc is complex and incompletely understood. The current view of this disease includes the development of vasculopathy, activation of the cellular and humoral immune responses, and progressive fibrosis. The pathological hallmark of SSc is an obliterative vasculopathy combined with interstitial fibrosis in target organs. Raynaud's phenomenon, a reversible process in primary disease, is due to dysregulation of the peripheral and autonomic nervous systems leading to vasospasm. However, in SSc, ischemia can progress due to irreversible changes within the endothelium associated with decreased production and responsiveness of endothelial derived vasodilators (nitric oxide and prostacyclins) and increased production and responsiveness of vasoconstrictors (endothelin-1). In addition, microvessels have increased permeability, increased leukocyte extravasation, and activation of coagulation and fibrinolytic factors with platelet aggregation, eventually leading to thrombosis. This vasculopathy affects capillaries, arterioles, and even large vessels and progresses to luminal occlusion due to intimal/medial hypertrophy and adventitial fibrosis with persistent endothelial damage and apoptosis. The process of revascularization is also defective in SSc, affecting both angiogenesis, in which new vessels arise from preexisting vessels, and vasculogenesis, in which new vessels derive from endothelial progenitor cells to replace damaged or senescent blood vessels, despite elevated levels of angiogenic factors. Therefore, in SSc there is a widespread obliterative vasculopathy associated with the failure to repair and replace damaged vessels, thus resulting in poor digital perfusion and the potential for ischemic digital loss [12].

## 5. Vascular Disease Distribution

Evidence has shown that both proximal and distal arteries are affected in SSc-related vasculopathy resulting in digital ischemic loss. Stafford et al. evaluated Doppler studies of arteries in the limbs, neck, and abdomen of 20 SSc patients compared with controls who were non-SSc rheumatology patients. They found that the ulnar arteries in SSc patients were significantly narrower (*P* = .002) and smoothly thickened (*P* < .0001) than those of non-SSc controls, while other arterial beds were not significantly different [13]. Another retrospective observational study analyzing brachial angiography found that 12 of 19 patients with SSc who exhibited Raynaud's phenomenon and digital ulceration had ulnar artery occlusion/stenosis with only 2 patients with radial artery involvement. Ulnar artery involvement was associated with the dcSSc subtype (*P* < .01) [14]. 

Hasegawa et al. studied macrovascular disease in patients with SSc with digital ulceration or gangrene using catheter arteriography of the upper and/or lower extremities. Seven of eight patients in this study were found to have macrovascular occlusion in the upper extremities. Of these, three had occlusion limited to digital arteries, three had obliteration of the ulnar and superficial palmar arch, and one had a radial artery occlusion. Of five patients who underwent lower extremity angiography, one had limited digital artery occlusion, one had occlusion of the posterior tibial artery, one had dorsalis pedis and arcuate occlusion, and two had occlusion of the plantar arch [15].

## 6. Clinical Evaluation

**Figure 1:**
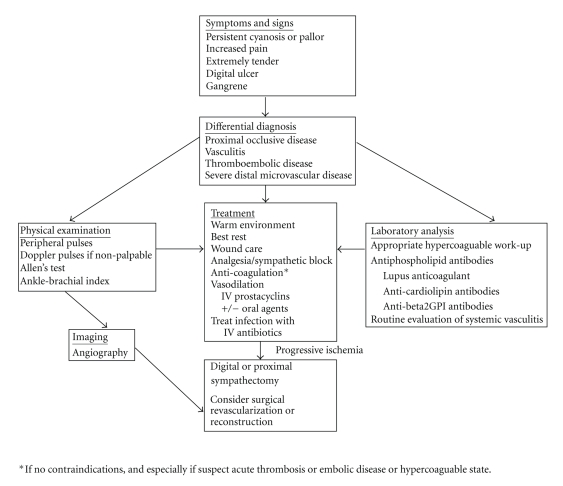
Algorithm for the evaluation and treatment of digital ischemic loss in systemic sclerosis.

The initial clinical evaluation for the signs of digital ischemia includes assessing for persistent discoloration (cyanosis or pallor), increased pain, digital ulceration, extreme tenderness, or frank gangrene. Nailfold capillaroscopic changes with dilatation, irregularity, megacapillaries, and drop-out are often present in SSc patients, and progression of these features may be predictive of the development of digital ischemia [16]. The differential diagnoses that should be considered in all connective tissue disease associated digital ischemia include proximal occlusive disease, vasculitis, thromboembolic disease, or severe distal microvascular disease. As such, all patients should be evaluated for peripheral pulses, dopplers if pulses are weak or nonpalpable, an Allen's test, and possibly ankle-brachial indices. Laboratory analysis for prothrombotic states including the antiphospholipid antibodies (lupus anticoagulant, anti-cardiolipin, and anti-beta2-glycoprotein I antibodies) should be performed in all patients. Prompt clinical evaluation and referral for treatment is critical to the prevention of progression to digital loss [17]; see Figure 1 for an evaluation and treatment algorithm.

## 7. Imaging

Angiographic techniques to evaluate for digital occlusions include conventional angiography, magnetic resonance angiography (MRA) or computed tomography (CT) angiography (Table 2). Conventional angiography is extremely sensitive for identifying stenosis, occlusion, aneurysm, or other vascular irregularities, but is invasive, involves high contrast load, and radiation exposure. MRA is noninvasive, and can visualize the vessel wall in addition to the lumen and surrounding structures. Spiral CT angiography allows 3-D imaging with shorter scanning times and without intravenous contrast or ionizing radiation.

The main indication for imaging is to identify proximal lesions amenable to angioplasty or surgery in cases of severe digital ischemia. It should be noted that MR and CT angiography are still investigational techniques and should be reserved for patients who have contraindications to conventional angiography. Conventional angiography is still considered the gold standard to visualize more distal arteries, especially if arterial reconstruction is being considered [12].

## 8. Treatment

Digit-threatening ischemia warrants prompt and aggressive treatment to control symptoms and prevent digital loss. As this is considered a medical emergency, hospitalization should be considered for all patients to expedite interventions. Nonpharmacologic treatments such as a warm environment and bed rest to decrease activity or possible injury to the affected limb are essential. A simple xeroform dressing with an antibiotic ointment can be used to prevent a superinfection and allow wound healing. Intravenous antibiotics should be used if overlying infection or osteomyelitis is suspected, especially in patients who are being considered for surgical debridement or if there is any collection of purulent material or necrotic tissue. Surgical debridement is generally reserved for patients with purulent drainage, necrotic/late stage ischemic tissue, or severe structural arterial disease who do not respond to medical therapies.

Analgesia with opioids is of utmost importance as pain due to critical digital ischemia is extremely intense. Local anesthetic blocks with lidocaine or bupivicaine without epinephrine may be helpful for pain control, but these interventions have not been studied in clinical trials. Temporary chemical sympathetic block should strongly be considered for patients with severe pain before surgical sympathectomy [12].

Anticoagulation is recommended for patients with rapidly advancing ischemic tissue who do not have contraindications. Intravenous heparin for 24–72 hours is typically used; however, no double-blind clinical trials have been performed [12]. Theoretically, this approach would be appropriate in cases where symptoms are suggestive of a new arterial occlusion thought to be due to an acute thrombosis or embolization. Further studies are needed to confirm the efficacy of this approach.

Aggressive vasodilatation is thought to improve bloodflow to ischemic areas. Firstly, oral calcium channel blocker doses should be titrated to maximum tolerated doses. However, intravenous prostacyclins, which not only vasodilate but also inhibit platelet aggregation, are considered the mainstay of management for acute digital ischemia. Intravenous epoprostenol or iloprost (0.5–2 ng/kg/min) daily infusions for 1–3 days, each infusion lasting 6 hours, is the recommended regimen. However, for patients with severe progressive ischemia, continuous prostacyclin infusion and higher doses may be required as tolerated. Studies have shown that intravenous iloprost was effective in reducing both the frequency and severity of ischemic attacks and in the healing of digital ulcerations [18, 19]. Epoprostenol was found to decrease the number of new digital ulcers in a double-blind trial in patients with SSc-associated pulmonary arterial hypertension [20]. Common side effects of these medications include hypotension, dizziness, headache, flushing, jaw pain, and gastrointestinal symptoms [21]. 

If symptoms are persistent and medical therapy fails, proximal or digital sympathectomy, microsurgical revascularization of the hand, and digital arterial reconstruction have been reported to improve digital vascular perfusion, heal digital ulcers, and substantially relieve or eliminate pain from one to 46 months postoperatively [22]. 

A proximal sympathectomy involves resection or ablation of a section of the cervical or thoracic sympathetic chain. This treatment is now only rarely performed due to its significant associated risks such as permanent Horner's syndrome, persistent neuralgia, and decreased localized cutaneous sweating; however, an endoscopic thoracic approach may be safer. Sympathectomy is less effective in patients with secondary than primary Raynaud's phenomenon and the duration and degree of improvement is variable [12].

Digital sympathectomy was introduced in the 1980s as an alternative to proximal sympathectomy. A recent study in 20 patients primarily with SSc, who had 42 ulcerated digits due to ischemia, found that periarterial sympathectomy led to complete healing or decrease in ulcer number in 28 of 42 digits after a mean of 96 months of follow-up. In addition, the rate of amputation in the treated patients with underlying autoimmune disease was half of that for patients with underlying atherosclerotic disease, indicating that patients with autoimmune-induced ischemia may be more amenable to treatment with digital sympathectomy [23]. However, controlled studies have not yet been performed, and given that the risk for perioperative complications is reported at 37%, with amputations and recurrent ulcerations at 14% and 18%, respectively, these procedures should be reserved for patients with severe ischemia and a threatened digit only after failure of vasodilator therapy [24]. In addition, these procedures should be performed by experienced hand or vascular surgeons at specialty centers. 

If there is larger vessel occlusive disease, especially at the level of the ulnar or radial artery, successful reconstruction can be performed with vein grafts [13]. Of 15 SSc patients with severe RP and digital ulceration and angiography-proven ulnar artery occlusion, no patients responded to conventional medical therapy; however, 8/15 patients underwent ulnar artery revascularization combined with digital sympathectomy and all subsequently experienced dramatic improvement in Raynaud's phenomenon and healing of digital ulcers [25]. 

A recent case report describes successful vascular reconstruction, with improved digital temperatures, pain and cold sensitivity, and health-related outcomes with peripheral artery bypass for vasoocclusive disease of the superficial palmer arch and tarsal arch in two SSc patients with severe digital ischemia [26]. Another case series noted that bypass surgery was successful in achieving early pain relief and ischemic wound healing for critical limb ischemia due to tibial artery occlusion in patients with SSc; however 4/8 of these patients subsequently experienced limb loss and 1 patient developed persistent recurrent ulcers [27]. Further studies particularly regarding the long-term outcomes of bypass surgery for the treatment of critical limb ischemia in SSc are warranted.

## 9. Conclusion

Systemic sclerosis is a complex autoimmune fibrosing disease with major vascular complications. Digital vasculopathy with critical ischemia is one of the more challenging complications and carries significant morbidity in a substantial proportion of patients. Identified risk factors for ischemic digital loss include the diffuse subtype of SSc, SSc specific antibodies (anti-centromere antibodies and anti-topoisomerase antibodies), current smoking, anti-beta2-glycoprotein I antibodies, and anti-granzyme B antibodies. Ischemic digital loss is related to both macrovascular disease with involvement of proximal and larger digital arteries, as well as microvascular involvement caused by neuroendothelial, structural, platelet, and procoagulant effects. The medical treatment algorithm for critical digital ischemia includes urgent evaluation for reversible causes, analgesia, intravenous vasodilator therapies, angiography, and possibly anticoagulation. Surgical therapies such as sympathectomies and vascular reconstructions are considered in patients who fail medical therapy. Further research is necessary to identify specific biomarkers for digital ischemia in patients with SSc, and to develop more effective treatments.

## Figures and Tables

**Table 1:** Potential risk factors for digital ischemic loss in patients with systemic sclerosis.

Likely	Possible
Diffuse cutaneous subtype	Anti-topoisomerase antibodies
Anti-centromere antibodies	
Current smoking	
Anti-beta2-glycoprotein I antibodies	
Anti-granzyme B antibodies	

**Table 2:** Radiographic considerations for evaluation of digital ischemic loss in patients with systemic sclerosis.

Modality	Pros	Cons
Conventional Angiography	Extremely sensitive for identifying vascular abnormalities	Invasive
		High contrast load
	Standardized technique	Radiation exposure
		Risk of inducing vasospasm

Magnetic Resonance Angiography (MRA)	Standardized technique	
	Noninvasive	
	No contrast load	Long scanning time
	No radiation exposure	Resolution inferior to CT or conventional angiography in distal digital vessels
	Can visualize vessel wall in addition to lumen	
	Can visualize surrounding structures	
	Can visualized venous lesions	

Computed Tomography (CT) Angiography	Noninvasive	Less contrast load than conventional angiography
	Excellent bone and soft tissue spatial relationships	Shorter scanning time
		Resolution inferior to conventional angiograpy in distal digital vessels
